# Monitoring of Antioxidant Efficacy of Mangrove-Derived Polyphenols in Linseed Oil by Physicochemical and Fluorescence Methods

**DOI:** 10.3390/antiox14020192

**Published:** 2025-02-07

**Authors:** Manjeet Singh, Eliot Botosoa, Romdhane Karoui

**Affiliations:** University of Artois, University of Lille, University of Littoral Côte d’Opale, University of Picardie Jules Verne, University of Liège, INRAE, Junia, UMR-T 1158, BioEcoAgro, F-62300 Lens, France; manjeet_singh@ens.univ-artois.fr (M.S.); herinirina.botosoa@univ-artois.fr (E.B.)

**Keywords:** mangroves, polyphenols, antioxidant, fluorescence, chemometry

## Abstract

This study was conducted to assess the antioxidant potential of polyphenolic extracts from *Rhizophora mucronata* and *Avicennia marina* as natural preservatives in comparison with synthetic butylated hydroxytoluene (BHT) and rosemary extract. Antioxidant activities were assessed by 2,2-diphenyl-1-picrylhydrazyl (DPPH), oxygen radical absorbance capacity (ORAC), and total phenolic content (TPC). Extracts were blended into linseed oil and evaluated for oxidative stability using a 15-day Schaal oven test. Physicochemical analyses, including peroxide value (PV), acid index (AI), p-anisidine value (p-AnV), and thiobarbituric acid reactive substances (TBARS), showed that mangrove-treated oils exhibited the highest stability against oxidation compared to the negative and positive controls. *R. mucronata* mature leaves presented the highest DPPH inhibition (93.40%) and the lowest TBARS value (0.33 ± 0.0 mg MDA/kg of oil) on day 11. Fluorescence spectroscopy provided complementary and valuable information. Statistical analysis using factorial discriminant analysis (FDA) achieved a classification accuracy of 91.43%, underlining the different oxidative profiles of the treated samples. These findings demonstrated the potential of extracts from mangrove plants as a sustainable alternative to synthetic antioxidants for food preservation. Future studies should explore broader food applications using advanced analytical techniques to optimize their efficiency and performing a series of toxicity evaluations.

## 1. Introduction

Located along tropical and subtropical coastlines, mangrove forests and plants are vital to coastal ecosystems. These salt-tolerant plants thrive in harsh environmental conditions, such as high salinity, nutrient-poor soils, and extreme temperatures [1]. To survive in these harsh environments, mangrove plants produce a variety of secondary metabolites, including polyphenols (such as flavonoids and tannins) and alkaloids, which not only help the plants to resist environmental stress but also exhibit strong bioactive properties. Among the various mangrove species, *Rhizophora mucronata* (*R. mucronata*) and *Avicennia marina* (*A. marina*) have gained considerable attention for their high concentrations of polyphenolic compounds that exhibit strong antioxidant and antimicrobial activities [2]. The selection of *R. mucronata* and *A. marina* was also driven by their ecological dominance in the mangrove ecosystems of Madagascar. Mangrove plants in this region remain poorly explored regarding their antioxidant properties compared to the well-studied mangrove species from the Asian mangrove belt. Their availability in Madagascar and the possibility of examining their antioxidant activities at different maturity stages (young and mature) made these plants the focus of this study. In addition, *R. mucronata* has been reported to have higher phenolic content in nutrient-rich regions, such as Indonesia [1], and *A. marina* is a variable species in response to salinity and ecological conditions found in Australian and Indian ecosystems [1]. Therefore, conducting research on these species within a wider context of the mangrove family may shed light on their unique contribution to antioxidant functionalities and facilitate comparative studies to understand the influence of geographical and climatic parameters.

Mangrove plant polyphenols, such as gallic acid, caffeic acid, and ellagic acid, possess more than one hydroxyl group, which enhances their ability to donate hydrogen atoms, thereby acting as potent neutralizers of lipid radicals and lipid chain oxidation initiators [2,3]. The flavonoids, such as quercetin and catechins, also act by stabilizing free radicals, and when combined with tannins and other phenolic acids, they exhibit synergistic antioxidant effects [2]. Such phenolic compounds help to enhance the oxidative stability by inhibiting hydroperoxide formation, delaying secondary oxidation and increasing shelf life [3]. Another benefit of mangrove plant polyphenolics is the diversity of their chemical structures; the bioactive compounds can chelate pro-oxidative metal ions like Fe^2+^ and Cu^2+^, resulting in the disruption of the Fenton reaction responsible for generating reactive oxygen species (ROS) [2]. While these chelating properties increase their antioxidant activity, trace metals in the extracts may sometimes produce pro-oxidant effects depending on the conditions [2,3]. This ambivalent behavior clearly justifies the need of further research to optimize the use of mangrove plant extracts so that they can express their full potential as a highly effective natural antioxidant, possibly equalling or even surpassing synthetic antioxidants in functionality.

The food industry has traditionally relied on synthetic antioxidants, such as butylated hydroxytoluene (BHT) and butylated hydroxyanisole (BHA), to prevent lipid oxidation in food products, particularly oils and fats [3]. However, growing concerns about the safety and long-term health effects of these synthetic preservatives have spurred interest in natural alternatives [4]. While rosemary and green tea extracts are well-established natural antioxidants, they have some drawbacks in terms of stability, extraction efficiency, and cost-effectiveness. On the other hand, mangrove-derived polyphenols, like those found in *R. mucronata* and *A. marina*, are rarely utilized in the food industry. Apart from *R. mucronata* and *A. marina*, other mangrove species, such as *Sonneratia alba*, *Bruguiera cylindrica*, and *Ceriops decandra*, also reveal excellent antioxidant potential due to their rich biochemical profiles [2]. For instance, *S. alba* contains flavonoids, terpenoids, and tannins, which contribute to its strong DPPH scavenging activity. Similarly, *B. cylindrica* is rich in phenolics and flavonoids that show high antiradical activity against DPPH and ABTS radicals. Likewise, *C. decandra* has strong radical scavenging activity, linked to the presence of polyphenolics [1]. Phenolic compounds, widely present in plants and agri-industrial by-products, exhibit significant antioxidant properties and play an essential role in enhancing food preservation [5]. Therefore, antioxidants derived from *R. mucronata* and *A. marina* are expected to provide a promising solution due to their strong antioxidant properties against lipid peroxidation, which is a primary cause of food spoilage. Studies have found that antioxidant molecules from mangroves may have a protective function against oxidative stress and thus can be used as a natural and sustainable alternative to synthetic antioxidants [2]. Likewise, flavonoids and phenolic acids of *A. marina* are also understood to inhibit peroxides formation and the secondary oxidation products in oils, supporting their importance as food shelf-life extenders alongside other biological functions [5]. Although tannins of *R. mucronata* are effective in inhibiting many prominent foodborne pathogens against *Escherichia coli* and *Salmonella* spp., extracts from *A. marina* have revealed a broad spectrum of antimicrobial as well as antioxidant properties in various food systems [1,6]. Despite these promising features, the potentials of mangrove-derived bioactive compounds as food preservatives are less explored, presenting an opportunity for research and development. Mangrove species have great potential for the future of food preservation due to an increasing consumer preference for natural, “clean label” foods and the growing demand for sustainable and safe preservatives.

With the high demand for natural and sustainable food preservatives and the promising bioactive properties of mangrove-derived compounds, it is essential to study the effectiveness of phenolic compounds in practical applications. Therefore, this study aimed to evaluate the antioxidant effect of leaf extracts from sub-Saharan mangrove plant species, specifically, *R. mucronata* and *A. marina* collected at the young and mature stages, on the oxidative stability of linseed oil. The main objective was to evaluate their potential as natural antioxidants and compare their effectiveness with that of BHT, used as a synthetic antioxidant, and rosemary extract selected as one of the most effective natural antioxidants and currently the most used in the food industry. Using advanced extraction and sophisticated analytical techniques, this study provides relevant scientific information on the ability of these natural extracts to control lipid oxidation in linseed oil, providing a sustainable and healthy alternative to synthetic preservatives.

Although previous studies have been carried out on mangrove plants, they are predominantly focused on theoretical evaluations. The present study aims to contribute to filling the gap by evaluating the antioxidant properties of bioactive molecules present in mangrove plants from sub-Saharan Africa, as well as their potential contribution to enhance food (e.g., seafood) sustainability in these regions. Therefore, this study combines biochemical/physiochemical analyses, including total phenolic content (TPC), DPPH inhibition, oxygen radical absorbance capacity (ORAC), and oxidative stability tests (acid index, peroxide value, p-anisidine value, and thiobarbituric acid reactive substances) with the use of a 2D fluorescence spectroscopy technique to monitor oxidation products and trace compounds, alongside practical applications in linseed oil treated with polyphenolic extracts from *R. mucronata* and *A. marina*.

## 2. Materials and Methods

### 2.1. Sample Collection and Location

**Figure 1:**
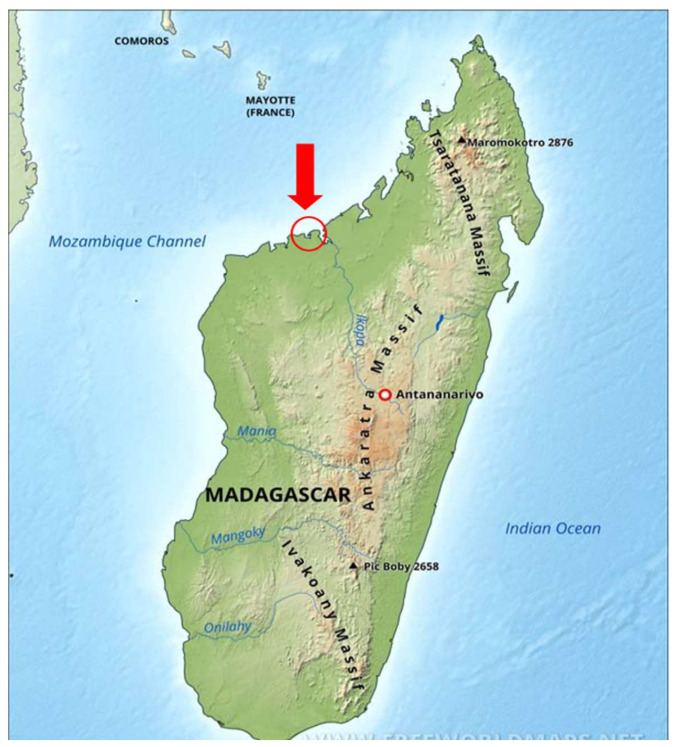
Location of mangrove leaf collection sites in Antrema, Madagascar. (Map of Madagascar sourced from Free World Maps, available at: https://www.freeworldmaps.net/africa/madagascar/map.html (accessed on 15 December 2024)).

In this study, underinvestigated mangrove species from Madagascar, specifically located along the sub-Saharan African coastline, were explored (Figure 1). The species selected for analysis include *A. marina* and *R. mucronata*, with both young and mature leaf samples of each collected species. Madagascar’s mangroves are vital ecosystems but remain relatively underexplored compared to other regions in the world. Mangroves play an essential role in coastal protection, biodiversity, and carbon sequestration, making them crucial for both ecological and environmental research.

The samples were collected from Antrema, located in Madagascar (geographically located between 15°43′ south latitude and 46°10′ east longitude) (Figure 1). The samples were collected between May and June 2023; the leaves were sun-dried just after collection from the field at ambient temperature (ranging from 30 to 33 °C). The leaves of *A. marina*, being thinner and less water-rich, required 2 days for complete dehydration, whereas the leaves of *R. mucronata*, presenting higher water content, took 4 days to dry completely. The leaves were spread in the thinnest possible layers and flipped every four hours to ensure uniform drying. Subsequently, they were oven-dried (Froilabo, model SP-BVEHF, France) at 35 °C for 9 days before being sent to France. Upon arrival in France, the leaves were oven-dried again (Froilabo, model SP-BVEHF, France) at 30 °C for 3 days to eliminate any remaining absorbed moisture that may have diffused into the leaves during transport. They were then stored in a dark, airtight condition until further analysis.

### 2.2. Chemical Analyses

Butan-1-ol, dichloromethane, 100% acetic acid, 95% denatured ethanol, n-Hexane, Folin–Ciocalteu reagent, gallic acid, 96-well microplate (opaque and transparent), 3,5-di-tert-butyl-4-hydroxytoluene (BHT), DPPH, phosphate buffer, isooctane, sodium thiosulphate pentahydrate, phenolphthalein 0.5 wt, % in ethanol/water (1:1), and TECAN (Spark^®®^ multimode microplate reader) was manufactured by Tecan Group Ltd. in Männedorf, Switzerland, and was supplied by Merck KGaA, located in Darmstadt, Germany. Potassium iodide (99% purity) and pure sodium hydroxide (97%) in pellet form were supplied by Labogros (Coueron, France). Rosemary extract was purchased from Danisco (Copenhagen, Denmark). Para-anisidine, sodium carbonate, and 2-thiobarbituric acid reagents were procured from Merck (Darmstadt, Germany). DMSO was obtained from Alfa aesar, Trolox from Cayman, and AAPH 2,2’-Azobis(2-amidinopropane) dihydrochloride) from Acros Organics (Hessen, Germany). Starch and chloroform were purchased from Roquette (Lestrem, France) and Carlo Erba (Val de Reuil, France), respectively. Fatty acid index (FAI), peroxide value (PV), para-anisidine value (p-AnV), and thiobarbituric acid TBARS measurements were directly performed on the linseed oil.

### 2.3. Defatting of Mangrove Leaves and Extraction of Phenolic Compounds

The dried leaves were ground with n-hexane (1:5, *w*/*v*) for 3 min using a laboratory blender (Waring, France) at ambient temperature. The resulting mixture was filtered using a coffee filter to separate the hexane solution from the leaf powder. This extraction step was repeated twice by adding fresh n-hexane solvent to the same defatted leaf powder and grinding for another 3 min each time. Defatted samples were then air-dried for 2 days under the fume hood to remove the remaining traces of n-hexane and then stored at −20 °C for further phenolic extraction and defatted powder to be used for Schaal oven test [7]. At first, a solvent mixture of 95% denatured ethanol, distilled water, and acetic acid (70:30:1) was prepared for extraction of phenolic compounds. A total of 4 g of defatted powder were mixed with 40 mL of the abovementioned solvent mixture and subjected to ultrasonic assisted extraction (UAE) (ELMA, Schmidbauer GmbH, Model TI-H-5, Singen, Germany) at 25 kHz and 60% power for 45 min. The extract was filtered through Whatman No. 4 filter paper. The remaining wet powder suspension (slurry) was subjected to a second extraction with a solvent mixture of ethanol, water, and acetic acid (90:10:1) under the same conditions. After the second extraction, the samples were centrifuged at 13,000 rpm for 10 min, and the liquid extract was collected and concentrated using a rotary evaporator at 38 °C [8]. The concentrated extract was stored in dark vials to avoid light-induced degradation and further purified by nitrogen evaporation for 5 h. This step resulted in a final product rich in phenolic compounds, which was then ready for analysis and for incorporation as a natural antioxidant in linseed oil used for the Schaal oven test.

### 2.4. Phenolic Content and Antioxidant Activity Measurements

#### 2.4.1. Total Phenolic Content (TPC)

TPC was determined by the Folin–Ciocalteu sodium carbonate method [9]. The reagent solution was prepared by mixing 1 mL of Folin–Ciocalteu reagent with 9 mL of distilled water, diluting it to 10%, and dissolving 1.06 g of sodium carbonate in 10 mL of distilled water (1 M). Gallic acid was used as the standard for calibration curve. A stock solution of 1 mg/mL gallic acid was prepared by dissolving 1 mg of gallic acid in 1 mL of ultra-pure water. From this stock solution, serial dilutions were made to obtain concentrations ranging from 100 µg/mL to 3.125 µg/mL. The extract solution for the samples was prepared as follows: 1 mg of extract was dissolved in 250 μL of dimethylsulfoxide (DMSO) to form the starting stock solution. This resulted in a concentration of 4 mg/mL. Further dilutions were made from this starting stock solution to obtain concentrations of 1 mg/mL and 0.5 mg/mL. The assay involved adding 50 µL of the extract or sample to a 96-well transparent microplate, followed by 50 µL of ultra-pure water, 50 µL of 10% Folin–Ciocalteu reagent, and 50 µL of 1 M sodium carbonate. The mixture was incubated, and the absorbance was measured at 650 nm. The phenolic content was quantified by comparing the absorbance of the sample with a standard curve of gallic acid.

#### 2.4.2. 2,2-Diphenyl-1-Picrylhydrazyl (DPPH)

The DPPH free radical scavenging test was used to evaluate the antioxidant capacity of samples such as *A. marina* (young and mature) and *R. mucronata* (young and mature). BHT was selected as a positive control. Firstly, BHT was prepared by dissolving 0.0010 g in 1 mL of ethanol. A 0.1 mM DPPH solution (VWR, France) was prepared by dissolving 0.0010 g of DPPH in 25 mL of ethanol, and a control solution (BHT) containing 1 mL of ethanol and 1 mL of DPPH was also prepared. The blank contained only ethanol. For example, stock solutions were prepared by dissolving 1 mg of each extract and positive control in 2 mL of ethanol. From this, serial dilutions were prepared to obtain the following concentrations: 100 µg/mL, 75 µg/mL, 50 µg/mL, and 25 µg/mL. Next, 150 μL of each extract and positive control solution was mixed with 50 μL of DPPH solution in a transparent 96-well microplate. The mixture was then incubated in the dark for 30 min, covered with aluminium foil. The absorbance of each sample and control was measured at 517 nm using a TECAN microplate reader [10]. The antioxidant activity of the samples was assessed by comparing their absorbance with that of the absorbance of the BHT control group. A lower absorbance value indicated a higher antioxidant activity. Finally, the percentage of DPPH inhibition was calculated using Equation (1):

(1) $$Inhibition\%=\frac{A_{control}-A_{sample}}{A_{control}}\times 100$$

where *A_sample_* is the absorbance of a sample solution and *A_control_* is the absorbance of the control solution containing all reagents except the test sample.

#### 2.4.3. Oxygen Radical Absorbance Capacity (ORAC)

The antioxidant capacity of the samples was determined using an ORAC assay, based on the method of Morel et al. [10] with some modifications. This assay measures the ability of antioxidant compounds to inhibit the loss of fluorescence in fluorescein (FL) induced by a peroxyl radical generator 2,2′-Azobis(2-amidinopropane) dihydrochloride (AAPH). The assay was carried out in a black, opaque 96-well microplate. The reaction mixture contained 100 μL of 75 mM phosphate buffer (pH 7.4), 100 μL of freshly prepared fluorescein solution (0.1 µM in phosphate buffer), 50 μL of freshly prepared AAPH solution (103.2 mg/mL in phosphate buffer), and 20 μL of sample per well. The samples were tested in triplicate and prepared at a concentration of 25 μg/mL, starting from a stock solution of 1 mg/mL in DMSO. Rosemary extract was tested at 12.5 μg/mL as a reference antioxidant, while Trolox was used as a standard at concentrations of 75, 50, 25, 12.5, and 6.25 µM. Chlorogenic acid (8.8 µM) was used as a quality control. The samples, fluorescein, and phosphate buffer were incubated at 37 °C for 10 min before starting the reaction by adding 50 μL of AAPH. The fluorescence was then measured every 5 min over a period of 70 min at an excitation wavelength of 485 nm and an emission wavelength of 535 nm using TECAN microplate reader.

The ORAC values were calculated by determining the area under the fluorescence decay curve (AUC) and comparing it to a Trolox standard curve. The results were expressed as micromoles of Trolox equivalents (TE) per milligram of dry extract (µmol TE/mg). The antioxidant capacity of the samples was determined relative to the standard.

### 2.5. Maceration and Oil Preparation for Schaal Oven Test

**Figure 2:**
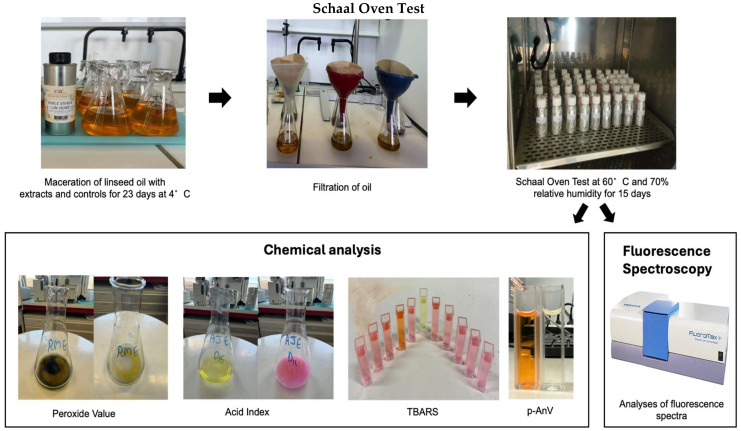
Schematic representation of linseed oil preparation, ageing, and analysis.

Two series of unrefined and cold-pressed linseed oil, collected from a French local plant oil factory (Huilerie de l’Orme Creux, Corbeuse, France) were mixed with extracts from *A. marina* (young and mature) and *R. mucronata* (young and mature), respectively. A total of 7 Erlenmeyer flasks (capacity 250 mL, VWR-France) were prepared, each containing 200 mL of linseed oil. Four of these Erlenmeyer flasks were supplemented with 20 mg of extract from *R. mucronata* (young and mature leaves) and *A. marina* (young and mature leaves). Additionally, three control flasks were prepared: a negative control (without additives), a positive control (containing 20 mg of BHT), and a control containing 20 mg of rosemary extract. After the addition of the extracts, the Erlenmeyer flasks were continuously stirred for 2 h using a magnetic stirrer at 600 rpm at 35 °C to ensure proper mixing. Due to the high viscosity of the leaf extracts, a challenge was encountered in achieving uniform solubility in linseed oil, a characteristic commonly observed in botanical extracts, as noted in a previous patent [11]. The viscosity hindered uniform solubility in linseed oil through simple mixing. Therefore, to address this issue, a maceration step was applied. The Erlenmeyer flasks were covered with aluminium sheets, sealed airtight, and stored at 4 °C for 23 days, ensuring that the extract was thoroughly solubilized in the oil [12]. After this period of maceration, the samples from each batch were filtered using a coffee filter to remove any solid residues (Figure 2). The filtered oils were then equally divided into glass bottles with 35 mL of oil in each bottle, ensuring equal headspace (15 mL) and a surface-to-volume ratio of 0.21 cm^2^/mL. The bottles were covered with aluminium foil and labelled with the type of sample and specific testing days. The samples were placed in a controlled environment (Froilabo, model SP-BVEHF, Collégien, France) at 60 °C with a relative humidity of 70% following Schaal’s oven test procedure. Oxidation levels in the oil were monitored and tested on day 0, day 3, day 7, day 11, and day 15 using standard analytical methods, including peroxide value (PV), para-anisidine value (p-AnV), and thiobarbituric acid reactive substances (TBARS).

#### 2.5.1. Fatty Acid Index

Fatty acid index (AI) was measured using a protocol adapted from Botosoa et al. [13], with some minor modifications. In this method, 2.5 g of linseed oil was dissolved in 20 mL of a binary solvent mixture consisting of ethanol and dichloromethane (1:1, *v*/*v*). The mixture was stirred at room temperature using a magnetic stirrer until the oil was completely dissolved. Two drops of phenolphthalein were then added as an indicator to signal the neutralization point in each sample. The solution was titrated with 0.01 mol/L sodium hydroxide solution until a persistent pink color appeared, indicating neutralization. The volume of sodium hydroxide required to reach this endpoint, known as the equivalent volume, was recorded. This equivalent volume was then used to calculate the fatty acid index, which quantifies the free fatty acids present in the oil and serves as an indicator of hydrolytic rancidity. The FAI value was expressed as milligrams of sodium hydroxide required to neutralize free fatty acids per gram of oil.

#### 2.5.2. Peroxide Value (PV) Measurements

Peroxide value (PV) was assessed using an iodometric titration method to quantify the concentration of hydroperoxides, which represent the primary oxidation products in linseed oils and provide an early indication of oxidative rancidity. In this procedure, 2.5 g of linseed oil was dissolved in 15 mL of a solvent mixture composed of glacial acetic acid and chloroform (3:1, *v*/*v*). To this mixture, 0.5 mL of potassium iodide was added, and the solution was stirred for one minute. Then 15 mL of distilled water was added, and the mixture was titrated with 0.01 M sodium thiosulfate in the presence of starch as an indicator (1%). The titration continued until the dark blue color disappeared, signaling the end point. The PV was expressed in milliequivalents (mEq) of oxygen per kilogram of linseed oil and reflects the degree of lipid peroxidation. This methodology was adapted from the protocol described by Botosoa et al. [13,14] with slight modification.

#### 2.5.3. p-Anisidine Value (p-AnV) Measurements

The p-AnV value determination was performed using the official AOCS method Cd 18–90 [15], intended to reveal secondary products of oxidation, primarily aldehydes such as 2-alkenals formed due to the breakdown of hydroperoxides. In this procedure, 1 g of linseed oil was dissolved in 25 mL of isooctane, and 5 mL of the resultant solution was mixed with 1 mL of p-anisidine which was prepared by dissolving 0.125 g of p-anisidine in 50 mL of glacial acetic acid. The absorbance of the solution at 350 nm was measured with a UV spectrophotometer (Model-2600, Shimadzu, Noisiel, France). The p-AnV was then determined using the formula given by AOCS. This test is indispensable to evaluate the extent of oxidation beyond the initial formation of hydroperoxides, as the reaction between p-anisidine and aldehydes forms a quantifiable complex, measurable by UV spectrophotometry. It provides important information about the oxidative stability of the oil.

#### 2.5.4. Thiobarbituric Acid Reactive Substances (TBARS)

The TBARS test was conducted by using a modified version of the method by Pokorný and Dieffenbacher [16]. In this procedure, 40 mg of oil sample was dissolved in 5 mL of 1-butanol, followed by the addition of 5 mL of TBA reagent (prepared by dissolving 0.2 g of thiobarbituric acid in 100 mL of 1-butanol). The mixture was then heated at 95 °C for 120 min in a Gesellschaft für Labortechnik (GFL) water bath (Model 1104, Burgwedel, Germany). After allowing the solution to cool by immersion in tap water for 10 minutes, the absorbance was measured at 530 nm using a UV spectrophotometer. The TBARS test measures malondialdehyde (MDA), a secondary oxidation product formed from the degradation of polyunsaturated fatty acids. MDA reacts with thiobarbituric acid to form a colored complex, which is quantified spectrophotometrically, providing an indication of the extent of oxidative degradation and the level of rancidity in the oil.

### 2.6. Fluorescence Measurements

Fluorescence spectra were obtained using a Fluoromax-4 spectrofluorometer (Jobin Yvon, Horiba, NJ, USA) at room temperature. The excitation radiation was set at an incidence angle of 60° to minimize reflected light, scattered radiation, and depolarization effects. Two spectra were recorded for each oil sample. Measurements were conducted at ambient temperature using a quartz cuvette of 1 cm path length, and the raw, unsmoothed data were captured. The excitation spectra were scanned over the ranges of 270–420 nm (for secondary oxidation products) with the emission wavelength set at 450 nm. The emission spectra (290–520 nm) for polyphenols were scanned with the excitation set at 270 nm. Corrections for instrumental distortions in the excitation and emission spectra were made using a rhodamine cell placed in the reference channel.

### 2.7. Statistical Analyses

For chemical analyses data, two-way ANOVA was carried out to assess the dispersion between different groups. Regarding the fluorescence measurements, principal component analysis (PCA) was applied separately to the normalized fluorescence spectra [17]. In a second step, factorial discriminant analysis (FDA) was performed on the 5 principal components (PCs) obtained from the PCA applied to each category of fluorophore (secondary oxidation products and polyphenols). The aim of this technique is to predict the membership of an individual to a pre-defined qualitative group [17]. Five groups were defined: day 0, day 3, day 7, day 11, and day 15. The comparison between the assigned group to the real group indicates the quality of the discrimination. Due to the low number of the investigated samples, FDA with cross validation was applied. The same samples were used both for model calibration and prediction. Finally, a concatenation approach was applied to the first 5 PCs of the excitation and emission spectra by combining the 5 PCs from the PCA of each fluorophore into a single dataset, aligning them sequentially, and applying the FDA in the same condition as indicated previously. ANOVA and FDA were performed with XLSTAT 2014 software (Addinsoft SARL USA, New York, NY, USA), while PCA was determined by using MATLAB version (R 2013b and 2014a) (The MathWorks, Natick, MA, USA).

## 3. Results and Discussion

### 3.1. Antioxidant Results

#### 3.1.1. Total Phenolic Content (TPC)

The TPC analysis shows that *R. mucronata* (at both young and mature stages) had the highest phenolic contents compared with both *A. marina* samples, with mature *R. mucronata* samples exhibiting the highest value, 191.22 mg GAE/g (Table 1).

Mature samples of both species appear to contain higher phenolic compound levels, although they are not statistically significant. Maturity contributes to an increase in phenolic content due to enhanced secondary metabolism as plants age [18,19]. In particular, mature leaves have had more exposure to environmental stressors, which trigger the production of phenolic compounds as part of the plant’s defence mechanism. When compared to the positive control, mature *A. marina* also showed a considerable amount of phenolic content, i.e., (103.61 mg GAE/g), which was significantly higher than the TPC observed in rosemary extract, approaching nearly twice its value. This result confirms the significant interest in phenolic compounds and the antioxidant activities of species collected from the mangrove ecosystem [20]. A recent study performed by Ismiel et al. [18] reported TPC of various mangrove leaves from Indonesia. The results suggested that leaves of *Sonneratia caseolaris* exhibited the highest TPC (182.89 mg GAE/g), followed by *R. mucronata* leaves with 125.10 mg GAE/g, which is apparently less than the TPC determined with *R. mucronata* leaves from Madagascar, i.e., 191.22 mg GAE/g. On the other hand, leaves of *A. marina* and *Rhizophora apiculata* were found to have lower TPC values of 83.02 mg GAE/g and 95.50 mg GAE/g, which is again less than the TPC measured in *A. marina* leaves from Madagascar, i.e., 103.61 mg GAE/g. No more details were provided about the age of either *A. marina* or *R. mucronata* samples reported in these studies, but leaf extracts from sub-Saharan African mangroves appear to show the highest TPC values. This difference may be due to the different geographical locations of the mangrove species. Otherwise, the TPC variation among different species emphasizes the influence of species-specific properties and phytochemical profiles on the phenolic content found in the leaves of mangrove. TPC is generally high in mangrove plants because they produce antioxidant phenolic compounds as part of their evolutionary defence against harsh environmental stress conditions, such as salinity. The ability to regulate salt through mechanisms like salt glands, leaf shedding, and ultrafiltration contributes to their phenolic abundance and strong antioxidant activity [18].

#### 3.1.2. DPPH Radical Scavenging Activity

BHT, the synthetic antioxidant (positive control), and the rosemary sample exhibited significantly lower antioxidant capacity, emphasizing the potent natural antioxidant potential of these mangrove species. The DPPH assay results highlight the strong antioxidant capacity of *A. marina* and *R. mucronata* when compared with BHT (Table 1). Mature *R. mucronata* exhibited the highest inhibition potential (93.40%) followed by young *R. mucronata* with 91.57%, in agreement with the findings of Luksamee et al. [21]. The antioxidant activity is likely enhanced in the mature samples due to the higher accumulation of secondary metabolites such as polyphenols, which naturally increase with plant age [18]. These compounds are known to scavenge free radicals effectively, explaining the superior activity of the mature stage. Their ability to donate protons and stabilize radicals highlights their significant role in antioxidant capacity [18]. In contrast, the young stage may contain lower concentrations of these antioxidants due to the metabolism related to their developmental stage. *A. marina* showed lower DPPH inhibition than *R. mucronata*, likely due to species-specific variations in their antioxidant profiles as indicated earlier. The lower activity of young and mature *A. marina* (81.94% and 80.47%, respectively) compared to *R. mucronata* may be due to differences in their secondary metabolite composition [19,20]. A study conducted by Thirunavukkarasu et al. [22] depicted a DPPH inhibition percentage of, respectively, 83.21% and 82.12% for the leaves of *A. marina* and *R. mucronata* found on the southeast coast of India. The slight variation in DPPH% between the sub-Saharan Africa and Indian mangrove leaves is likely due to environmental factors like stress conditions, salinity, soil, and climate. These factors could add to the production of phenolic compounds accordingly [1,2].

#### 3.1.3. ORAC Assay

The ORAC assay results indicate a strong antioxidant capacity across all samples containing mangrove plant extracts, with mature *A. marina* showing the highest ORAC value (306.67 µmol TE/g) and (306.53 µmol TE/g) for the mature extract of *R. mucronata* (Table 1). Typically, maturity contributes to greater scavenging of free radicals. This higher ORAC value in mature plants reflects their ability to neutralize peroxyl radicals, which increases with a higher content of phenolic compounds found in mature tissues [18]. Maturity is associated with increased biosynthesis of antioxidant compounds, driven by the plant’s need to defend against environmental stressors like UV radiation, herbivory, and pathogens. The young leaf extract for both species showed reduced ability to neutralize peroxyl radicals. The significant ORAC values in *R. mucronata* species further reinforce the contribution of these bioactive compounds in neutralizing oxidative stress. The lowest ORAC values obtained for the control, such as rosemary, highlight the superior antioxidant capacity of mangrove species in the present study. As the mangrove plants thrive in ecologically challenging zones, their robust antioxidant mechanism operates through the accumulation of phenolic compounds and plays a potential role in countering the oxidative stress caused by harsh environmental stress conditions like salinity [1].

### 3.2. Primary Oxidation Products: Acid Index (AI) and Peroxide Value (PV)

#### 3.2.1. Acid Index (AI)

The AI values obtained during the Schaal oven test provided detailed information about the oxidation degradation behavior of the linseed oil treated with various antioxidants, including leaf extracts of *A. marina* and *R. mucronata* (young and mature), the synthetic antioxidant (BHT), and the natural antioxidant (rosemary). Based on these results, the AI, PV, TBA, and p-AnV of all samples on day 11 can be considered as the best reference to compare the level of free fatty acids generated by lipolysis and the production of secondary oxidation products. The AI values obtained particularly for day 11 reflect the comparative effectiveness of various antioxidants in mitigating oxidative degradation of linseed oil during the Schaal oven test (Table 2). 

On day 11, the negative control (Neg) showed an AI value of 2.32 ± 0.22 mg NaOH/g attributed to natural hydrolytic and oxidative changes in the absence of antioxidants. This value is relatively high and represents auto-oxidation of PUFAs, such as α-linolenic acid, under accelerated conditions [23]. The synthetic antioxidant, BHT, acting as a positive control showed a much lower AI value of 2.05 ± 0.01 mg NaOH/g on day 11 of the experiment, reflecting its ability to delay the formation of free fatty acids more effectively than the negative control. This superior performance of BHT is due to its efficient chain-breaking mechanism, where it donates hydrogen atoms to lipid radicals, effectively inhibiting the propagation step of oxidation [23]. On the other hand, the natural extract of rosemary (ROS) showed a similar AI of 2.08 ± 0.01 mg NaOH/g, reflecting its strong antioxidative power. This effect may be explained by the synergistic interaction of phenolic compounds in rosemary, supplementing its main antioxidative effects [24,25]. However, the slight difference between rosemary and BHT in the AI context could also be linked to rosemary’s reduced ability to suppress free fatty acid formation under extended oxidative stress.

Among the mangrove extracts, *A. marina* young (AY) and *A. marina* mature (AM) showed AIs of 2.09 ± 0.01 mg NaOH/g and 2.06 ± 0.02 mg NaOH/g, respectively, on day 11, which falls within the range of a moderate antioxidant effect (Table 2). *A. marina* mature (AM) performed slightly better in terms of antioxidant activity, probably due to higher concentrations of polyphenols in mature leaves [1]. *R. mucronata* young (RY) and *R. mucronata* mature (RM) both showed AI values of 2.12 ± 0.0 mg NaOH/g, exhibiting the same antioxidative action during this period. The comparable AI values for *R. mucronata* young (RY) and *R. mucronata* mature (RM) may result from the interaction of bioactive compounds, particularly tannins, which stabilize hydroperoxides early in the oxidation process. However, slight variations in AI could be due to different degradation rates of these bioactive molecules, influenced by the oil matrix’s composition and oxidative environment [3]. This early stabilization trend in *R. mucronata* mature throughout this study implies more consistent antioxidative activity, possibly due to its richer tannin content and other bioactive compounds [26]. In general, mangrove leaf extracts, especially young and mature *A. marina* (AY & AM), showed better control over hydrolytic degradation when compared with samples containing *R. mucronata* extracts, rosemary extract, and negative control.

#### 3.2.2. Peroxide Value (PV)

The PV provides key information about the linseed oil’s oxidative stability during the Schaal oven test, particularly under various antioxidant treatments (Table 2). Regarding the negative control (Neg), PV increased sharply from 7.78 ± 0.2 meq O_2_/kg oil on day 0 to 50.42 ± 0.8 meq O_2_/kg oil on day 11, highlighting the extent of oxidative degradation in the absence of antioxidant treatment. This value represents the sensitivity of PUFAs, such as α-linolenic acid, to peroxidation, which is favored by heat and oxygen exposure in the headspace of the linseed oil samples [23]. The significant increase observed up to day 11 highlights the formation of primary oxidation products, hydroperoxides, which is consistent with linseed oil’s PUFA-rich composition [27]. The positive control showed an enhanced oxidative stability, with a PV of 34.94 ± 0.25 mEq O_2_/kg on day 11. Compared to the negative control, the early peroxide formation indicates that BHT is effective in scavenging free radicals [3]. Rosemary extract (ROS) showed a PV of 48.52 ± 0.31 mEq O_2_/kg on day 11, similar to the negative control. This result suggests that although rosemary extract has potent phenolic antioxidants, with time it loses its activity, probably due to the exhaustion of primary antioxidants and generation of secondary oxidative products. The higher PV in the rosemary-treated oil compared to the mangrove-treated oils may be attributed to its less effective metal-chelating ability, as phenolic compounds like rosmarinic acid primarily act as radical scavengers rather than chelators [2,5].

Among all the studied mangrove extracts, *R. mucronata* mature (RM) showed a PV of 36.19 ± 0.27 mEq O_2_/kg on day 11 (Table 2). Mangrove plant leaves, with their high content of phenolics and tannins, are thought to provide very strong interruption in the chain reactions of lipid peroxidation and a chelating effect toward pro-oxidative metals [3]. A similar trend was seen in *R. mucronata* young (RY), which reached a PV of 33.55 ± 0.02 mEq O_2_/kg on day 11. However, the slightly earlier and lower accumulation of peroxides in *R. mucronata* young (RY) indicates its higher antioxidant capacity compared to *R. mucronata* mature (RM). Both *A. marina* mature (AM) and *A. marina* young (AY) exhibited significant antioxidative properties but were slightly less effective than *R. mucronata* extracts. By day 11, *A. marina* mature (AM) and *A. marina* young (AY) had attained PVs of 30.94 ± 0.27 mEq O_2_/kg and 34.39 ± 0.55 mEq O_2_/kg, respectively. This highlights the crucial role of flavonoids and polyphenols, which are present in higher concentrations in mature leaves, contributing to their enhanced antioxidant capacity [18]. The slightly higher PV in *A. marina* young (AY) compared to *A. marina* mature (AM) may reflect the earlier depletion of less concentrated bioactives in younger leaves under oxidative stress [2]. The results obtained for PV underline the higher antioxidant potential of mangrove extracts, especially those from *A. marina* in comparison with synthetic (BHT) and natural (rosemary extract) antioxidants. These results underline the potential of mangrove-derived antioxidants as natural additives to improve the oxidative stability of PUFA-rich oils such as linseed oil. These results also demonstrate that mangrove extracts were able to control the peroxide formation better than the rosemary extract, thus highlighting their potential.

### 3.3. Secondary Oxidation Products: Thiobarbituric Acid Reactive Substances (TBARS) and p-Anisidine Value (p-AnV)

#### 3.3.1. TBA Results

The TBARS values of the various extracts indicate the antioxidative efficiency in the retardation of secondary oxidation in linseed oil (Table 2). The negative control (Neg) had a TBARS value of 0.50 ± 0.0 mg MDA/kg on day 11, reflecting the production of secondary oxidation products due to the lack of protection by antioxidants. The overall higher TBARS levels emphasize uncontrolled oxidative degradation. The positive control in this case, BHT, had a slightly lower TBARS value of 0.43 ± 0.0 mg MDA/kg, indicating its competency in suppressing lipid peroxidation at this stage. BHT’s effectiveness is attributed to its radical scavenging activity, which interrupts the oxidation chain reactions. However, the slightly higher TBARS values compared to mangrove leaf extracts may reflect BHT’s limited ability to stabilize secondary oxidation products [3]. The drop in TBARS by day 11 illustrates BHT’s ability to prevent secondary oxidation even under prolonged oxidative stress [28]. Rosemary extract (ROS) showed a TBARS value of 0.62 ± 0.0 mg MDA/kg, slightly higher than that of the synthetic control. This reflects rosemary’s phenolic compounds ability to neutralize free radicals and delay secondary oxidation while being less robust than BHT under extended oxidative conditions. This weaker performance under prolonged stress is likely due to the exhaustion of primary antioxidants like carnosic acid, which is more effective in the early stages of oxidation [25].

Of all the mangrove extracts, the extracts from *R. mucronata* mature (RM) and *R. mucronata* young (RY) presented the best antioxidative activities. *R. mucronata* mature (RM) showed a TBARS value of 0.33 ± 0.0 mg MDA/kg, indicating a high antioxidative activity (Table 2). The superior performance of *R. mucronata* mature (RM) is attributed to its high tannin content, which efficiently inhibits the decomposition of hydroperoxides into malondialdehyde and other secondary products [1,3]. The decline after the initial spikes testifies to the contribution of tannins and flavonoids in inhibiting hydroperoxide decomposition. In fact, *R. mucronata* young (RY) showed 0.29 ± 0.0 mg MDA/kg TBARS, the lowest value among all samples, and reflected the excellent performance of this extract. Extracts from *A. marina* mature (AM) and *A. marina* young (AY) also presented good antioxidative performance on day 11, with values of 0.42 ± 0.0 mg MDA/kg and 0.41 ± 0.0 mg MDA/kg, respectively. The comparable values for *A. marina* mature (AM) and *A. marina* young (AY) suggest effective radical scavenging activity but indicate slightly higher values compared to *R. mucronata* extracts, reflecting the relatively lower chelating capacity of flavonoids. These results highlight their ability to stabilize oxidation, although they were slightly less effective than *R. mucronata* extracts. The observed difference in performance may be due to the concentration of polyphenols and the potential pro-oxidant behavior of the extract in oil [29].

#### 3.3.2. p-AnV Results

p-AnV values provide insights into the performance of various antioxidants against the formation of secondary oxidation products, mainly aldehydes, in linseed oil under oxidative stress (Table 2). The negative control had a p-AnV value of 10.91 ± 0.11 on day 11, showing a strong accumulation of secondary oxidation products resulting from a lack of protection by an antioxidant. This higher value of p-AnV underscores the high susceptibility of linseed oil, rich in polyunsaturated fatty acids (PUFAs), to hydroperoxide decomposition and aldehyde formation. In fact, a high C18:3 linolenic acid content of linseed oil accelerates its oxidation, creating unsaturated aldehydes much faster [30]. The positive control (BHT) recorded a slightly higher p-AnV of 10.96 ± 0.01 on day 11, indicating no conclusive suppression of secondary aldehyde formation compared to the negative control sample. This result can be explained by the partial depletion of BHT’s chain-breaking antioxidant capacity under prolonged oxidative stress, leading to the accumulation of aldehydes despite its initial radical scavenging activity [3]. The earlier spike in p-AnV on day 7 highlights the ability of BHT to interrupt oxidation chains, although its radical scavenging capacity appears to be overwhelmed under prolonged oxidative stress. The gradual decrease in p-AnV towards later stages suggests a limited but sustained antioxidative performance. The lower p-AnV in samples with rosemary extract compared to the negative control highlights the contribution of its phenolic compounds such as rosmarinic acid in delaying aldehyde formation. However, the exhaustion of primary antioxidants by day 11 likely reduced its efficacy in mitigating secondary oxidation products [25]. Rosemary extract, ROS, showed a p-AnV of 9.13 ± 0.16 on day 11 which is slightly lower than that of the negative control, evidencing moderate antioxidative activity mainly due to its phenolic compounds such as rosmarinic acid. Rosemary was able to delay the beginning of aldehyde formation, but in later stages, its antioxidative potential was reduced, leading to higher p-AnV values at that stage [25].

Among the mangrove leaf extracts *A. marina* mature (AM) and *A. marina* young (AY), *A. marina* mature (AM) showed a p-AnV of 11.75 ± 0.04, showing an unexpected antioxidative performance at this step, although it was less effective compared to *R. mucronata* extracts (Table 2). The higher p-AnV in *A. marina* young (AY) compared to *A. marina* mature (AM) suggests that the lower concentration of polyphenols in young leaves might have contributed to the insufficient suppression of aldehyde formation under oxidative conditions. Additionally, the thermal degradation of phenolic compounds in *A. marina* young (AY) likely exacerbated aldehyde production [3]. This behavior evidences, as discussed above, the action of polyphenols in delaying hydroperoxide decomposition, which is partially limited under prolonged storage. In linseed oil, the high unsaturation level accelerates oxidation. Even antioxidants with strong radical scavenging activity might not fully suppress the formation of secondary oxidation products due to the overwhelming oxidative burden [31]. *A. marina* young (AY) had the highest p-AnV of 13.6 ± 0.1 on day 11, underlining its diminished potency in suppressing aldehyde formation compared to mature leaf extracts. The increased value indicates the possibility of thermally stressed and degraded phenolic compounds in young leaves contributing to secondary oxidation phenomena in the case of corresponding extracts [32,33]. *R. mucronate mature* (RM) attained lower antioxidative performance as confirmed by its p-AnV of 12.85 ± 0.14 on the 11th day, illustrating the production of secondary oxidation products. The high depletions observed afterwards (day 15) provides strong evidence for this result in terms of longevity during long-term aldehyde mitigation. On the other hand, *R. mucronata* young (RY) exhibited a slightly lower p-AnV of 11.66 ± 0.07 in samples containing mangrove extracts, which reflects its higher antioxidative activity compared to *R. mucronate* mature (RM). This trend is probably due to its high phenolic compound contents, such as tannin [34]. The difference in performance between young and mature extracts highlights the importance of bioactive compound concentrations in determining antioxidant efficacy. This may also be attributed to the higher bioavailability of hydrolysable tannins in *R. mucronata* young (RY), which actively scavenge radicals and delay hydroperoxide decomposition, leading to lower aldehyde accumulation [2].

### 3.4. Fluorescence Excitation Spectra of Primary and Secondary Oxidation Products in Linseed Oil

The study conducted by Rabiej and Szydłowska-Czerniak et al. [35] demonstrated that fluorescence spectroscopy can identify minor oxidation products and bioactive compounds that might not be noticeable using chemical tests. In the current study, results showed that FI in areas related to oxidation products was higher in oil enriched with extracts compared to the control samples, even though the chemical analysis indicated comparatively low PV and TBARS for oil with mangrove extract samples. Likewise, the referenced study observed an increase in FI in areas caused by newly developed fluorescent oxidation products, even when antioxidant-enriched oils chemically exhibited better oxidative control. This overlapping indicates that FI does not just show the level of oxidation but also indicates the continued presence of compounds like tocopherols and polyphenols or their breakdown products like degradation intermediates. The findings reinforce that fluorescence spectroscopy provides a sensitive and complementary approach to monitoring both oxidation dynamics and antioxidant interactions with oils, extending beyond the scope of conventional chemical methods and thus emphasizing the utility of fluorescence for understanding the complex interplay between antioxidants and oxidation processes.

**Figure 3:**
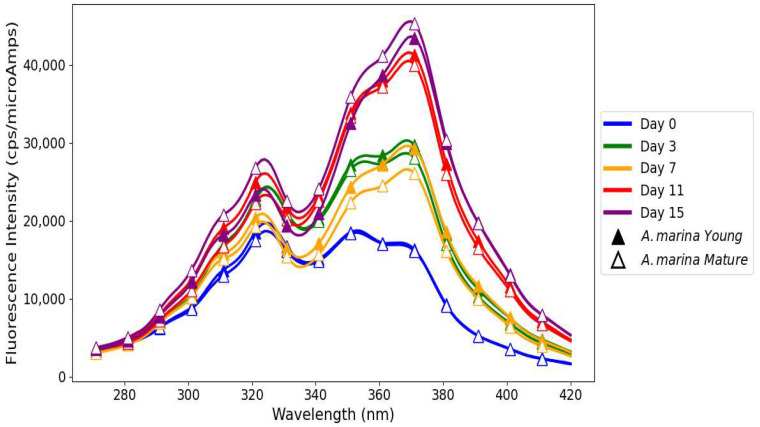
Excitation fluorescence spectra of linseed oil samples containing young (▲) and mature (△) *A. marina* extract obtained after emission set at 340 nm.

**Figure 4:**
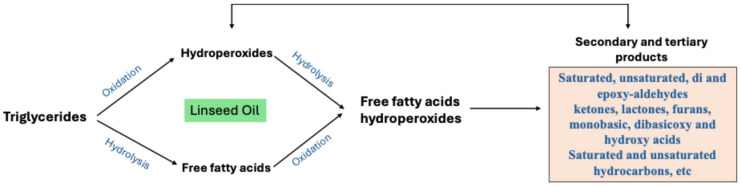
Oxidative and hydrolytic degradation reaction pathways in linseed oil. (Adopted from Gharby et al. [37]).

Linseed oil when treated with *A. marina* extracts displayed different oxidative stability profiles for young and mature leaves (Figure 3), reflecting differences in their phenolic compositions. The FI of both primary and secondary oxidation products increased consistently from day 0 to day 15 in linseed oil treated with young *A. marina* extract. This trend indicates the progressive development of oxidative processes: hydroperoxides are formed at the early stages of oxidation and accumulate over time due to the decrease in the antioxidant capacity of the extract [36,37]. At all the time points, most FIs for secondary oxidation products were higher than those of primary oxidation products. This evidences that while the extract can provide some inhibition to early-stage oxidation, it is not able to suppress the accumulation of the secondary products such as aldehydes and ketones, which account for rancidity and off-flavors in oils [38]. The diagram (Figure 4) shows the mechanism of triglycerides being broken down by the oxidative and hydrolytic degradation pathways in linseed oils, which lowers the quality of the oil. When oxygen reacts with unsaturated fatty acids, unstable hydroperoxides are created. These hydroperoxides break down into secondary products like aldehydes, ketones, and other volatile substances. These substances give oils unpleasant smells, rancidity, and undesirable flavors due to the accumulation of the secondary oxidation products aldehydes, ketones, etc., which detract from their nutritional value and sensory appeal [37]. Additionally, FIs of both primary and secondary oxidation products in oil treated with young *A. marina* extract are consistently higher compared to the negative control, positive control, and rosemary extract. This indicates that the suboptimal antioxidant efficacy of the extract may further lead to poor oxidation control, particularly during the later stages. A study conducted by Michotte et al. [38] indicated that phenolics such as caffeic acid and catechin rapidly degrade during the oxidation process, which may result in diminished effectiveness and pro-oxidant actions under heated conditions. This underscores the importance of the interplay between phenolics and oil components, their degradation behavior, and environmental factors like concentration and temperature in determining whether they act as antioxidants or pro-oxidants.

In contrast, linseed oil when treated with mature *A. marina extract* illustrated superior oxidative stability, attributed to the enhanced antioxidant potential of mature leaves [26] (Figure 3). The FIs of primary and secondary oxidation products were almost balanced on day 0, showing the effective suppression of oxidation. On day 3, both peaks rose significantly, revealing an insufficient scavenging of radicals and the conversion of hydroperoxides due to the possible presence of minor undetectable oxidation products in the extract mixture [35,39]. A slight reduction in FI on day 7 showed the stabilization effect of the bioactive compounds in mature *A. marina* such as proanthocyanidins and flavonoids. However, a sharp resurgence on day 11 highlighted the significant accumulation of secondary oxidation products, indicating the extract’s diminishing efficacy in controlling advanced oxidation [40]. By day 15, the FIs for both primary and secondary products reached their peak, reflecting the extract’s limited prolonged antioxidative action. A study by Sotler et al. [40] found that the pro-oxidant behavior of polyphenols arises from their interaction with trace metals (e.g., Fe^2^⁺, Cu^2^⁺), which catalyze reactive oxygen species generation via the Fenton reaction, and their dual redox nature. This effect is influenced by concentration, with low levels being insufficient to scavenge radicals and higher levels reducing metals to propagate oxidation. Environmental factors like pH, oxygen, and co-antioxidant availability further dictate their antioxidant or pro-oxidant role.

**Figure 5:**
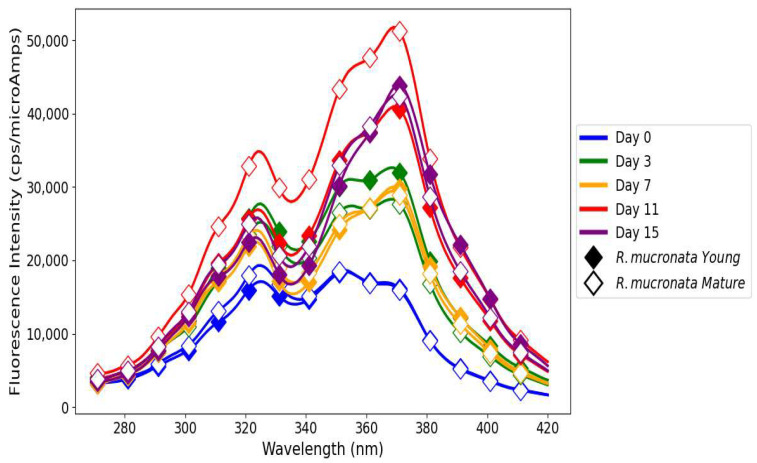
Excitation fluorescence spectra of linseed oil samples containing young (♦) and mature (◇) *R. mucronata* extract obtained after emission set at 340 nm.

On day 0, oil treated with *R. mucronata* young leaf extract had balanced antioxidant activity; thus, both FIs were equal in magnitude for the primary and secondary oxidation products, as depicted in Figure 5. By day 3, radical scavenging and hydroperoxide stabilization were very strong, and both FIs had notably increased, showing the very effective early-stage antioxidative action. On day 7, a high secondary oxidation peak reflected continued advanced oxidation, whereas a drop in primary oxidation products showed the breakdown of antioxidants. On day 11, a temporary stabilization was observed with a recovery of primary oxidation products, which may be due to the reactivation of hydrolysable tannins, while secondary oxidation products continued to accumulate. On day 15, the FI of secondary oxidation products reached its peak, while primary oxidation products stabilized at a lower level. This tendency highlights the inability of *R. mucronata* young leaf extract to suppress advanced stages of oxidation for a longer period [41].

The linseed oil when treated with mature *R. mucronata* extract showed the highest FI, indicating very low or no control over the formation of primary and secondary oxidation products. This unexpected behavior may be due to the presence of heavy metal traces or unknown fluorophore oxidation product formation during the oxidation process [35] (Figure 5). On day 0, mature *R. mucronata* extract effectively suppressed hydroperoxides and secondary oxidation products. However, FIs of both the primary and secondary oxidation products increased on day 3, reflecting a strong antioxidative activity at the early stage. The situation was then followed by sustained but insufficient antioxidant action under oxidative stress by day 7. On day 11, the sharp rise in FIs of both primary and secondary oxidation products underlined the presence of pro-oxidant compounds, likely influenced by the interactions between polyphenols and trace metals such as Fe^3^⁺. The dual behavior of polyphenols, depending on their molecular structure and oxidation conditions, contributed to the propagation of radicals instead of their scavenging [42]. However, while the FI of primary oxidation products slightly decreased on day 15, the FI of secondary products continuously increased, indicating the extracts’ inability to control the advanced stages of oxidation [43].

**Figure 6:**
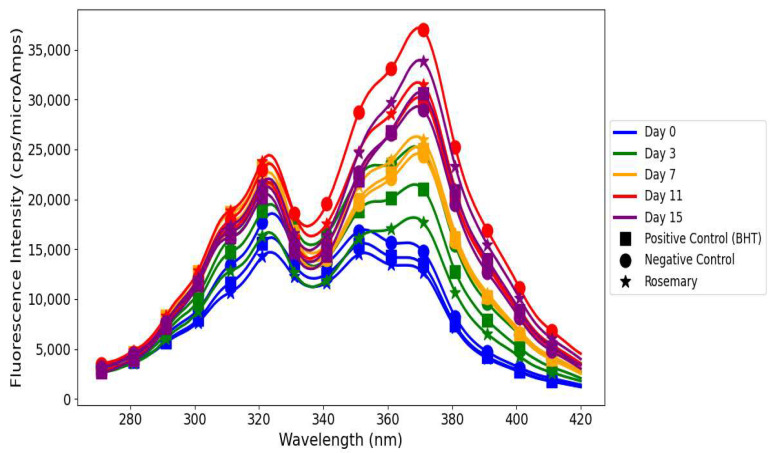
Excitation fluorescence spectra of linseed oil samples containing positive control—BHT (■), negative control—linseed oil without added antioxidants (●), and rosemary extract (★) obtained after emission set at 340 nm.

The negative control showed a rapid accumulation of primary and secondary oxidation products over time (Figure 6). Untreated linseed oil had the highest FI, with the following trend: from day 0 to day 7, it was FI of NEG > POS > rosemary, and on day 11 FI of NEG > rosemary > POS. This trend corresponds to the fast development of oxidation because of the absence of antioxidants. By day 15, a decline in FIs indicated oxygen consumption, thereby marking the advanced stages of oxidation dominated by aldehydes and ketones [44]. BHT-treated linseed oil, POS (Figure 6), showed lower FIs than the negative control mostly throughout the storage period, thereby providing better oxidative stability. While FIs were lower than those of rosemary-treated samples initially (day 11 FI of rosemary > POS), they stabilized by day 15, reflecting BHT’s capacity to delay the onset of oxidation but exhibiting signs of antioxidant depletion as oxygen levels declined [45]. Rosemary extract exhibited (Figure 6) an intermediate antioxidant performance with sustained suppression of oxidation products. Its FIs were consistently lower than those of the negative control but surpassed those of BHT on day 11. By day 15, a decrease in primary oxidation product FIs and stabilization of secondary oxidation product FIs reflected oxygen depletion and the partial exhaustion of rosemary’s phenolics like carnosic acid and rosmarinic acid, which delayed but did not entirely prevent advanced oxidation [25,46].

The differences in results between chemical analyses and fluorescence spectroscopy in the determination of lipid oxidation are indicative of the different aspects of oxidation dynamics measured by each technique. Chemical analyses including PV, TBARS, and p-AnV quantify oxidation products, while fluorescence spectroscopy provides additional information about primary and secondary lipid oxidation products as well as minor bioactive compounds containing conjugated double bonds [35]. In this study, FI increased in mangrove-treated linseed oils for oxidation products, which may point out the presence of bioactive polyphenols, such as flavonoids and tannins, but also potential degradation intermediates. On the other hand, chemical analyses showed better oxidative stability in mangrove-extract-treated linseed oil, evidenced by reduced PV and TBARS compared to controls, except for p-AnV, which was higher for mangrove-extract-treated linseed oils. This discrepancy suggests differences in sensitivity and specificity of these analytical techniques, in agreement with previous findings. For example, Sikorska et al. [47] monitored the degradation of tocopherols and pheophytins alongside the formation of new fluorescent oxidation products during the storage of rapeseed oil using fluorescence spectroscopy, as also observed by Rabiej and Szydłowska-Czerniak et al. [35]. These studies clearly show that fluorescence spectroscopy is very sensitive to minor and previously undetectable compounds, which might not be identified by conventional chemical analyses. This increases the value of fluorescence spectroscopy as a complementary analytical tool for extending our understanding of the complex interactions between antioxidants and oxidation products.

Rosemary-treated linseed oil showed a clear reversal in FI on day 11, which can be attributed to its dynamic redox cycling mechanism, which may regenerate antioxidant potential via intermediate quinones that facilitate dynamic electron transfer processes [48]. Furthermore, the redox cycling coupled with trace metal chelation may also suppress secondary oxidation products and stabilize FI [49]. In contrast, mangrove polyphenols were equally effective in depressing primary and secondary oxidation as evidenced by the chemical assays. However, they lacked a regeneration mechanism, resulting in higher FIs due to their intrinsic fluorophores and the build-up of minor oxidation products, which in this case were not revealed by the chemical tests. This sensitivity to additional oxidation dynamics further underscores the complementary nature of fluorescence spectroscopy. The mangrove-treated linseed oils initially showed higher FI (~20,000 cps/microAmps) compared to controls (~15,000 cps/microAmps), which was indicative of the intrinsic fluorescence of bioactive polyphenols and the formation of oxidation intermediates. These results agree with previous observations that fluorescence spectroscopy does not measure oxidation directly but reflects the presence of residual antioxidants together with their breakdown products, providing deeper insight into the lipid oxidation mechanism. While chemical analyses confirmed the antioxidant efficacy of mangrove extracts in mitigating lipid oxidation, fluorescence spectroscopy revealed additional dynamics.

### 3.5. Emission Fluorescence Spectra of Polyphenols in Linseed Oil

**Figure 7:**
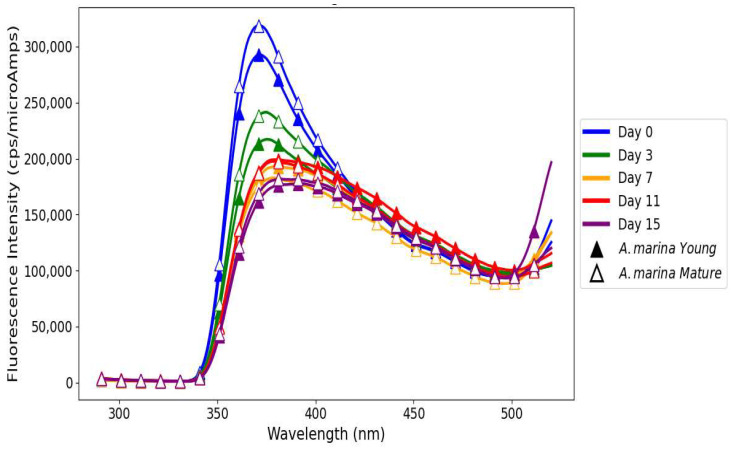
Emission fluorescence spectra of linseed oil samples containing young (▲) and mature (△) *A. marina* extract obtained after excitation set at 270 nm.

For young and mature *A. marina* extracts mixed with linseed oil, the highest values of FI were obtained on day 0 for both samples, indicating very high initial antioxidant capacity. By day 15, the values of FI obtained for both samples were the lowest and showed the normal progress of oxidation, with time depleting the antioxidant compounds (Figure 7). In general, *A. marina* extract with a higher stage of maturity had the highest FI on both on day 0 and day 3, respectively, because of its higher tannin- and flavonoid-resistant phenolic contents capable of scavenging reactive oxygen species and breaking the chain reaction of oxidation [39]. This antioxidant behavior contrasts with the more erratic trends observed in *R. mucronata*. For samples aged 11 days, FI increased from day 7, especially for the mature *A. marina* extract. Such an inverse tendency may indicate oxidative instability or a secondary release of phenolic compounds, and there is a correlation with such observations in the trends of peroxide values.

**Figure 8:**
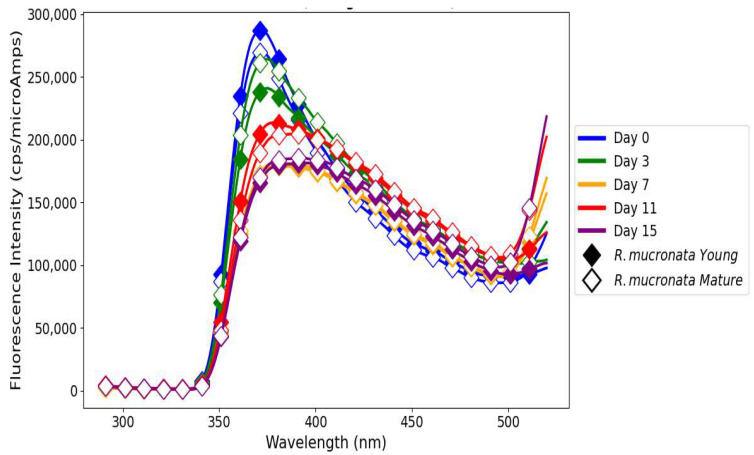
Emission fluorescence spectra of linseed oil samples containing young (♦) and mature (◇) *R. mucronata* extract obtained after excitation set at 270 nm.

The examination of fluorescence of linseed oil with *R. mucronata* extracts (young and mature), shown in Figure 8, revealed differences in oxidative stability, highlighting the contrast between phenolic compounds found in young and mature leaves of this plant species. Linseed oil with *R. mucronata* extracts on day 0 had high initial antioxidant activity. When comparing young leaves with mature leaves on day 0, young *R. mucronata* showing the highest FI. On day 3, the reduction in FI of both samples showed an antioxidant action. However, the mature *R. mucronata* extract showed higher stability than the young extract during this period, reflecting the persistence of mature leaf phenolics to scavenge radicals [1]. This trend continued as the FI decreased further on day 7, when the young extract had shown significant development of oxidation processes characterized by the formation of secondary products such as aldehydes and ketones [30]. On day 11, the FI of both samples increased, suggesting a reactivation of antioxidants. The young *R. mucronata* extract showed higher antioxidative stability with slower development of oxidation, while the mature extract showed signs of antioxidant exhaustion after prolonged stress. On day 15, the FI of both samples decreased, but the decrease was more pronounced in the case of the young extract than that of the mature one. This further justifies the better performance of the mature *R. mucronata*. The phenolics of *R. mucronata* effectively counteract lipid radicals and bind pro-oxidant metals, thereby slowing down oxidative processes [26].

**Figure 9:**
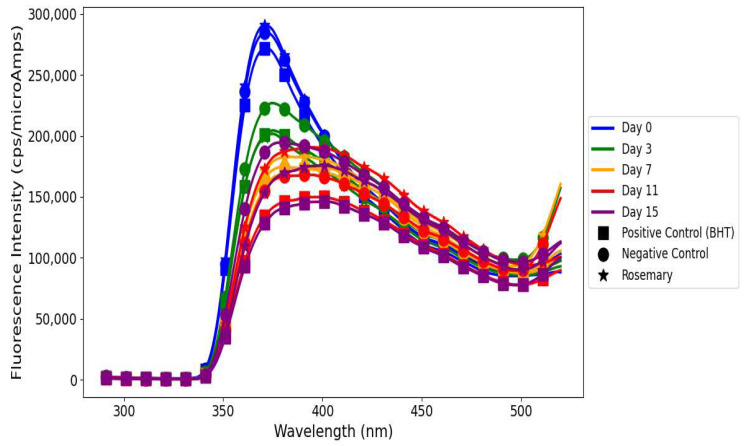
Emission fluorescence spectra of linseed oil samples containing positive control—BHT (■), negative control—linseed oil without added antioxidants (●), and rosemary extract (★) obtained after excitation set at 270 nm.

All the samples showed a reduction in FI with increased storage time, proving the continuing oxidative degradation of the oils over time. In particular, this trend for the positive control (linseed oil treated with BHT) shows that its FI continued to drop through the storage time, evidencing its antioxidant effect, though it was limited in longer storage periods. The untreated linseed oil used as the negative control (Figure 9) exhibited the highest initial FI on day 0, which was likely due to the presence of endogenous polyphenols such as phenolic acids and flavonoids [50]. The gradual reduction in the FI by day 15 shows the likely build-up of oxidation products and the breakdown of initial fluorophores due to the absence of external antioxidants that could prevent lipid oxidation from occurring. This pattern is consistent with the findings of Frankels et al. [51], who indicated that untreated oils experience increased decay when subjected to heat stress. The rosemary extract showed FI values (Figure 9) within the same range as those of mangrove plant extracts, namely, *A. marina* young and mature and *R. mucronata* young and mature, underlining their equal antioxidant potential. Among the samples, although all samples demonstrated a decrease in values, rosemary extract and mangrove samples showed better oxidative stability compared to the positive control. While a moderate decline was observed on day 7, the slight recovery on day 11 suggests the sustained activity of rosemary’s phenolic compounds, such as rosmarinic acid, in mitigating oxidative degradation [52,53]. Such a performance may reflect the residual activity of polyphenolic compounds from rosemary and bioactive polyphenols from the mangrove extracts [25]. Starting on day 11, however, the rosemary extract showed a unique inversion as its FI value started to slightly recover towards renewed antioxidant activity. There was no such phenomenon observed in either the negative (untreated linseed oil) or the positive (BHT) controls as the FI consistently declined, unhampered by any stabilization or reversal process. More importantly, the comparison of negative and positive control samples with linseed oil treated with natural extracts of rosemary and mangrove underlines the value addition these natural antioxidants confer. Linseed oil blended with natural extracts showed increased oxidative stability in contrast to the negative control, which showed a rapid oxidative degradation, and the positive control, which exhibited a gradual but steady decline from day 11 to day 15. This result emphasizes the potential of mangrove polyphenols to act as effective natural antioxidants capable of delaying the lipid oxidation process and thereby maintaining oil stability under storage conditions.

### 3.6. Chemometric Tools Applied to the Fluorescence Spectra

#### 3.6.1. Excitation Fluorescence Spectra Obtained After Emission Set at 450 nm

In this study, fluorescence spectroscopy was employed to monitor the formation of oxidation products in stored samples at five distinct time points, i.e., day 0, day 3, day 7, day 11, and day 15. The goal was to investigate how ageing influenced the development of oxidation products and affected the sample composition. To better differentiate between the time points, PCA and FDA techniques were applied. The results obtained from PCA allowed for slight discrimination between samples as a function of storage time. Therefore, to enhance the separation between the five time points, FDA was applied, and the similarity map provided a clear distinction between the groups.

**Figure 10:**
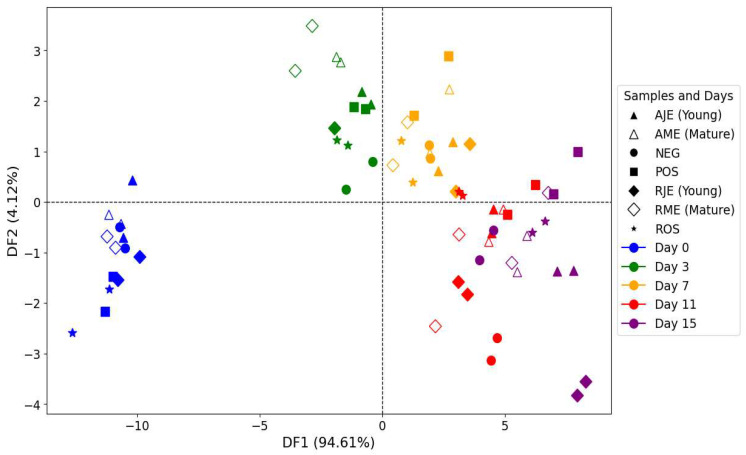
Factorial discriminant analysis similarity map of cross validation applied to the first 5 PCs of the PCA performed on emission spectra acquired after emission set at 450 nm determined by discriminant factors DF1 and DF2 for the ageing period (day 0 (blue), day 3 (green), day 7 (yellow), day 11 (red), and day 15 (purple)). AJE (▲) represents *A. marina young*, AME (△) represents *A. marina mature*, RJE (◆) represents *R. mucronate* young, RME (◇) represents *R. mucronata mature*, POS (■) is the positive control—BHT, NEG (●) is the negative control—linseed oil without added antioxidants, and ROS (★) represents rosemary extract.

In the FDA plot (Figure 10), Discriminant Factor 1 (DF1) explained 94.61% of the variance, showing the most significant distinction between samples as a function of ageing days. Samples from day 0 (blue) were distinctly clustered on the far left, while those of day 15 (purple) were positioned in the lower-right quadrant, indicating substantial changes in oxidation product profiles as ageing progressed. Samples belonging to day 3 and day 7 were positioned between day 0 and day 15, suggesting a gradual accumulation of oxidation products over time. Day 11 samples clustered closer to day 15, indicating that by day 11, the oxidation processes had already significantly altered the sample composition. However, the affiliation of ROS samples aged 11 days in the cluster of samples aged 7 days seemed to suggest that their oxidation product profiles were similar, confirming their effectiveness in retarding the oxidation process.

The cross-validation table (Table 3) underscores the FDA model’s efficacy in classifying samples across various time points, achieving an overall correct classification of 85.71%. The model performed exceptionally well for early storage days, with day 0 and day 3 both achieving perfect classification rates of 100%. This high accuracy reflects the distinct chemical profiles of oxidation products in these initial stages, where early-stage oxidation markers are clearly distinct. The group belonging to day 7 showed a slightly lower but still high classification accuracy of 92.86% compared to previous days, indicating that the model continues to effectively differentiate oxidation products at this intermediate stage. The minor overlap observed here may signal the onset of gradual changes in the oxidation profile as oxidative reactions progress.

In contrast, samples belonging to day 11 and day 15 demonstrated a lower correct classification percentage, with 71.43% for day 11 and 64.29% for day 15. These reduced accuracy percentages suggest an increasing overlap in oxidation profiles at these later stages, likely due to the stabilization of secondary oxidation products. As oxidation reaches more advanced stages, the rate of new product formation declines, resulting in similar profiles for days 11 and 15 that are harder to distinguish. This pattern aligns with known oxidation behavior in oils, where oxidation tends to plateau over time and distinct changes in oxidation products become less pronounced. The FDA model captures the early oxidative phases well, but the convergence of oxidation profiles in later stages reduces the classification percentage, particularly between days 11 and 15, where the stabilization of oxidation products complicates differentiation.

#### 3.6.2. Emission Fluorescence Spectra Acquired After Excitation Set at 270 nm

At first, PCA was applied to examine the overall trends in the data, but while some clustering was apparent, the groups lacked definitive and clear separation. The FDA applied to the first five PCs results significantly improved the differentiation between time points, providing a better view into the progressive oxidation of polyphenols.

**Figure 11:**
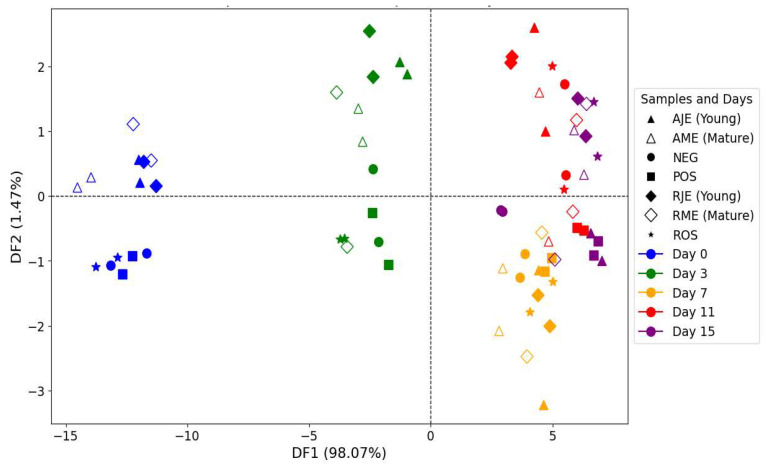
Factorial discriminant analysis similarity map of cross validation applied to the first 5 PCs of the PCA performed on emission spectra acquired after excitation set at 270 nm determined by discriminant factors DF1 and DF2 for the ageing period (day 0 (blue), day 3 (green), day 7 (yellow), day 11 (red), and day 15 (purple)). AJE (▲) represents *A. marina young*, AME (△) represents *A. marina mature*, RJE (◆) represents *R. mucronate* young, RME (◇) represents *R. mucronata* mature, POS (■) is the positive control—BHT, NEG (●) is the negative control—linseed oil without added antioxidants, and ROS (★) represents rosemary extract.

The FDA plot (Figure 11) clearly illustrates how the polyphenolic effect changed over time. Discriminant Factor 1 (DF1), which accounted for 98.07% of the total variance, was the dominant axis of separation, showing a clear shift from day 0 to day 15. The grouping showed that on day 0, samples were clustered in the far-left region of the plot, reflecting their relatively intact polyphenolic profile at an early stage. In contrast, on day 15, samples were positioned on the extreme right in purple, indicating their degradation over time due to these mechanisms that diminish their effectiveness, thus confirming the substantial chemical changes that occur throughout the time period. The intermediate time points, such as day 3 and day 7, showed a gradual progression, with each time point moving further away from day 0 as oxidation increased. Interestingly, day 11 and day 15 overlapped, suggesting that after day 11, the oxidation rate stabilizes, leading to fewer changes between these two later time points. Discriminant Factor 2 (DF2) accounted for 1.47% of the total variance and contributed to the fine-tuning of the group separation, though it had less influence than DF1.

The cross-validation table (Table 4) suggests the FDA model’s effectiveness in distinguishing between polyphenol profiles at different time points. With an overall correct classification rate of 77.14%, the model exhibits robust differentiation for early storage days, achieving 100% classification accuracy for days 0 and 3. This high rate indicates that the polyphenolic profiles of these early time points are chemically distinct, aligning with initial antioxidative activity and compositional stability in these samples. For day 7, the model’s classification accuracy decreases to 85.71%, suggesting some overlap in polyphenol characteristics as storage progresses. This moderate accuracy, however, still reflects sufficient discrimination at this intermediate stage, possibly due to ongoing but less pronounced changes in the polyphenolic structure. In contrast, samples belonging to day 11 and day 15 displayed lower classification scores of 50% each, signifying considerable overlap between these later stages. This overlap indicates a stabilization of polyphenol degradation or transformation, as the oxidative changes begin to plateau after day 7. Consequently, the biochemical differences between days 11 and 15 become less discernible, complicating accurate classification.

**Figure 12:**
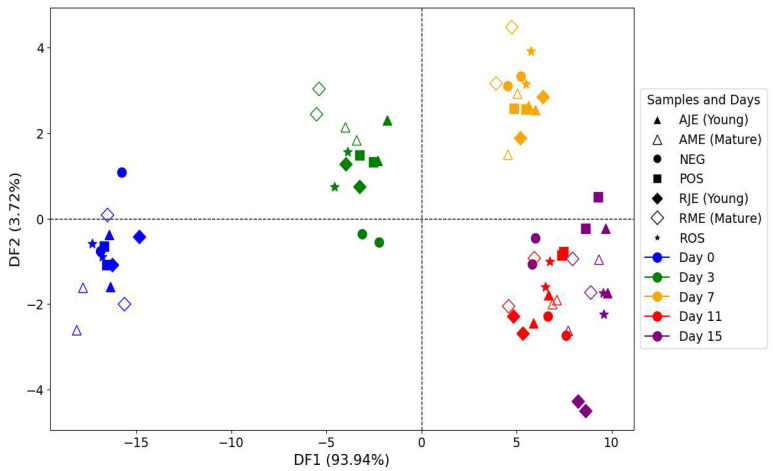
Factorial discriminant analysis similarity map of cross validation applied to the first 5 PCs of the PCA performed on emission and excitation spectra set at 270 and 450 nm, respectively, and determined by discriminant factors DF1 and DF2 for the ageing period (day 0 (blue), day 3 (green), day 7 (yellow), day 11 (red), and day 15 (purple). AJE (▲) represents *A. marina young*, AME (△) represents *A. marina mature*, RJE (◆) represents *R. mucronate* young, RME (◇) represents *R. mucronata* mature, POS (■) is the positive control—BHT, NEG (●) is the negative control—linseed oil without added antioxidants, and ROS (★) represents rosemary extract.

In order to simultaneously take into account all of the information contained in the polyphenol and secondary oxidation products fluorescence spectra, a concatenation approach was applied by combining the first 5 PCs of the PCA applied on each fluorophore. The map of the cross-validation dataset, defined by the first two discriminant factors of the FDA, is shown in Figure 12. Considering discriminant factor 1 (FD1) accounts for 93.94% of the total variance, a clear discrimination between samples as a function of storage time was observed.

These results suggest that the concatenation of the fluorescence datasets may be a potential approach for recognizing samples according to their storage time. Indeed, a correct classification with cross-validation was observed for 91.43% (Table 5).

## 4. Conclusions

This study highlights the significant potential of mangrove-derived extracts from *R. mucronata* and *A. marina* as natural antioxidants for enhancing the oxidative stability of linseed oil. The chemical analyses showed their ability to decrease primary and secondary oxidation products under accelerated storage conditions. At times, their performance even surpassed that of synthetic BHT and rosemary extract. Fluorescence spectroscopy complemented these findings by allowing deeper insights into the dynamics of oxidation including the detection of fluorescent intermediates and their interactions with trace metals that could not be captured by conventional physicochemical tests. Additionally, chemometric tools such as FDA applied to the oxidation products achieved 85.71% correct classification while that of polyphenols achieved 77.14% correct classification. These results demonstrate the dual role of mangrove plant extracts in mitigating oxidation while facilitating advanced classification techniques, offering eco-friendly alternatives to synthetic preservatives. This study opens the way to new approaches in food technology, addressing consumer demand for natural, sustainable, and health-conscious food preservation strategies by integrating fluorescence data and chemometric modeling. However, the lack of sensory and toxicity data represents a limitation for further application. This study has laid a foundation, but future research using other advanced analytical techniques, such as mid-infrared spectroscopy, ^1^H and/or ^13^C nuclear magnetic resonance, and chromatographic techniques including high-performance liquid chromatography (HPLC) and gas chromatography–mass spectroscopy (GC-MS) could help to understand the bioactive compounds in these extracts more comprehensively. Moreover, the current findings should encourage further investigation into their potential toxic effects before their real-world application in food formulation dedicated to human consumption.

## Figures and Tables

**Table 1:** Comparative antioxidant activities of leaf extracts from mangrove plants and controls (BHT and rosemary extract) based on TPC, DPPH, and ORAC.

Sample	TPC (mg GAE/g of Dry Extract)	DPPH Inhibition (%)	ORAC (μmol TE/g of Dry Extract)
Rosemary (control)	51.45 ± 21.70 ^C^	56.21 ± 4.27 ^C^	128.21 ± 38.03 ^C^
BHT (positive control)	ND	59.74 ± 2.14 ^C^	ND
*A. marina* (young)	92.75 ± 9.28 ^B^	81.94 ± 5.36 ^B^	288.52 ± 2.59 ^A^
*A. marina* (mature)	103.61 ± 9.34 ^B^	80.47 ± 5.20 ^B^	306.37 ± 4.53 ^A^
*R. mucronata* (young)	188.84 ± 19.70 ^A^	91.57 ± 0.98 ^A^	236.64 ± 9.49 ^B^
*R. mucronata* (mature)	191.22 ± 21.70 ^A^	93.40 ± 1.86 ^A^	306.53 ± 21.45 ^A^

Standard deviations are expressed as mean ± SD, indicating variability within each group. Different superscript letters denote significant differences between groups (*p* < 0.05), meaning that values sharing the same letter are not significantly different, while those with different letters represent statistically significant differences between samples based on Fisher test. Abbreviations: oxygen radical absorbance capacity (ORAC) (n = 2), 2,2-diphenyl-1-picrylhydrazyl radical scavenging activity (DPPH) (n = 3), total phenolic content (TPC) (n = 3), butylated hydroxytoluene (BHT), Trolox equivalent (TE), gallic acid equivalent (GAE), and not determined (ND),

**Table 2:** Comparative physicochemical analysis of linseed oil supplemented with mangrove leaf extracts (*R. mucronata* and *A. marina*—young and mature stages) and control samples (rosemary, positive control (linseed oil supplemented with BHT), and negative control (linseed oil without added antioxidants)). Assessment of AI, PV, TBARS, and p-AnV.

Sample Name	Stage	AI (mg NaOH/g of Linseed Oil)	PV (mEq O_2_/kg of Linseed Oil)	TBARS	p-AnV
Neg D0	(Control)	2.01 ± 0.02 ^H^	7.78 ± 0.28 ^N^	1.66 ± 0.00 ^E^	1.94 ± 0.04 ^Q^
Neg D3	(Control)	2.40 ± 0.02 ^ABC^	24.58 ± 0.25 ^H^	0.56 ± 0.00 ^S^	3.74 ± 0.30 ^NO^
Neg D7	(Control)	2.35 ± 0.02 ^BCD^	44.73 ± 0.54 ^C^	1.46 ± 0.01 ^G^	11.18 ± 0.02 ^D^
Neg D11	(Control)	2.32 ± 0.22 ^BCDE^	50.42 ± 0.85 ^A^	0.5 ± 0.00 ^V^	10.91 ± 0.11 ^D^
Neg D15	(Control)	2.19 ± 0.02 ^CDEFGH^	16.40 ± 0.00 ^M^	0.47 ± 0.00 ^Z^	4.13 ± 0.06 ^N^
Pos D0	(Control)	2.22 ± 0.02 ^CDEFGH^	6.97 ± 0.28 ^N^	2.75 ± 0.01 ^A^	0.36 ± 0.05 ^T^
Pos D3	(Control)	2.31 ± 0.07 ^BCDEF^	23.39 ± 0.28 ^H^	0.82 ± 0.00 ^Q^	6.5 ± 0.24 ^HI^
Pos D7	(Control)	2.26 ± 0.01 ^BCDEFG^	34.72 ± 0.55 ^E^	1.1 ± 0.00 ^N^	12.46 ± 0.12 ^B^
Pos D11	(Control)	2.05 ± 0.01 ^GH^	34.94 ± 0.25 ^E^	0.43 ± 0.00 ^AA^	10.96 ± 0.01 ^D^
Pos D15	(Control)	2.21 ± 0.01 ^CDEFGH^	24.97 ± 0.27 ^L^	0.4 ± 0.00 ^W^	5.7 ± 0.21 ^J^
Ros D0	(Control)	2.58 ± 0.58 ^A^	7.58 ± 0.57 ^ST^	1.16 ± 0.01 ^L^	1.33 ± 0.00 ^R^
Ros D3	(Control)	2.21 ± 0.00 ^CDEFGF^	19.19 ± 0.00 ^O^	0.6 ± 0.00 ^L^	3.99 ± 0.10 ^NO^
Ros D7	(Control)	2.38 ± 0.01 ^ABC^	32.17 ± 0.82 ^H^	1.75 ± 0.00 ^D^	8.44 ± 0.07 ^F^
Ros D11	(Control)	2.08 ± 0.01 ^GH^	48.52 ± 0.31 ^B^	0.62 ± 0.00 ^S^	9.135 ± 0.16 ^E^
Ros D15	(Control)	2.19 ± 0.01 ^CDEFGH^	27.13 ± 0.55 ^K^	0.51 ± 0.00 ^Y^	5.45 ± 0.19 ^JK^
AY D0	(Young)	2.1 ± 0.02 ^FGH^	6.58 ± 0.84 ^T^	1.22 ± 0.00 ^K^	2.49 ± 0.34 ^P^
AY D3	(Young)	2.22 ± 0.01 ^CDEFGH^	20.79 ± 0.55 ^N^	0.85 ± 0.00 ^O^	5.33 ± 0.12 ^JK^
AY D7	(Young)	2.25 ± 0.03 ^BCDEFG^	30.73 ± 0.59 ^I^	2.01 ± 0.00 ^C^	10.78 ± 0.01 ^D^
AY D11	(Young)	2.09 ± 0.01 ^GH^	34.39 ± 0.55 ^EF^	0.41 ± 0.00 ^AB^	13.6 ± 0.11 ^A^
AY D15	(Young)	2.19 ± 0.02 ^CDEFGH^	23.53 ± 1.11 ^M^	0.33 ± 0.00 ^AB^	5.62 ± 0.04 ^JK^
AM D0	(Mature)	2.11 ± 0.01 ^EFGH^	4.0 ± 0.56 ^U^	1.12 ± 0.00 ^M^	5.23 ± 0.21 ^KL^
AM D3	(Mature)	2.27 ± 0.01 ^BCDEFG^	17.54 ± 0.57 ^PQ^	1.1 ± 0.00 ^P^	3.6 ± 0.04 ^O^
AM D7	(Mature)	2.4 ± 0.02 ^ABC^	29.18 ± 0.02 ^J^	1.59 ± 0.00 ^F^	7.92 ± 0.01 ^G^
AM D11	(Mature)	2.06 ± 0.02 ^GH^	30.94 ± 0.27 ^I^	0.42 ± 0.00 ^AC^	11.75 ± 0.04 ^C^
AM D15	(Mature)	2.19 ± 0.02 ^CDEFGH^	20.34 ± 0.01 ^N^	0.74 ± 0.00 ^R^	6.47 ± 0.22 ^HI^
RY D0	(Young)	2.12 ± 0.01 ^EFGH^	3.4 ± 0.28 ^U^	1.27 ± 0.00 ^H^	0.87 ± 0.17 ^S^
RY D3	(Young)	2.27 ± 0.00 ^BCDEFG^	18.74 ± 0.51 ^O^	1.24 ± 0.00 ^J^	4.88 ± 0.23 ^LM^
RY D7	(Young)	2.46 ± 0.01 ^AB^	33.19 ± 0.57 ^G^	0.77 ± 0.00 ^R^	12.52 ± 0.12 ^B^
RY D11	(Young)	2.12 ± 0.00 ^EFGH^	33.55 ± 0.02 ^FG^	0.29 ± 0.00 ^AE^	11.66 ± 0.07 ^C^
RY D15	(Young)	2.22 ± 0.02 ^CDEFGH^	16.97 ± 0.28 ^QR^	0.43 ± 0.00 ^Z^	6.86 ± 0.17 ^H^
RM D0	(Mature)	2.14 ± 0.00 ^DEFGH^	3.19 ± 0.56 ^U^	1.26 ± 0.00 ^I^	4.69 ± 0.12 ^M^
RM D3	(Mature)	2.31 ± 0.00 ^BCDEF^	16.2 ± 0.28 ^R^	0.62 ± 0.00 ^U^	6.38 ± 0.13 ^I^
RM D7	(Mature)	2.38 ± 0.02 ^ABC^	30.18 ± 0.27 ^I^	2.52 ± 0.00 ^B^	11.86 ± 0.08 ^C^
RM D11	(Mature)	2.12 ± 0.01 ^EFGH^	36.19 ± 0.27 ^D^	0.33 ± 0.00 ^AD^	12.85 ± 0.14 ^B^
RM D15	(Mature)	2.19 ± 0.00 ^CDEFGH^	18.39 ± 0.55 ^OP^	0.59 ± 0.00 ^X^	2.83 ± 0.05 ^P^

Evolution of chemical parameters of linseed oil during storage at 60 °C and 70% relative humidity. Means within a column sharing the same letter (^A–Z^) are not significantly different (*p* > 0.05) based on Fisher’s LSD test, while different letters indicate significant differences (*p* < 0.05). Values represent means of two samples (n = 2). Abbreviations: *A. marina* young (AY), *A. marina* mature (AM); *R. mucronata* young (RY), *R. mucronata* mature (RM), negative control (Neg) (linseed oil without added antioxidants), positive control (Pos)—butylated hydroxytoluene (BHT), rosemary extract (Ros). D0, D3, D7, D11, and D15 are the storage and sample testing days. Key chemical tests include acid index (AI), para-anisidine value (p-AnV), peroxide value (PV), and thiobarbituric acid-reactive substances (TBARS).

**Table 3:** Classification table of factorial discriminant analysis with Leave-One-Out Cross Validation of linseed oil during ageing using excitation fluorescence spectra obtained after emission set at 450 nm.

Observed/Predicted	Day 0	Day 3	Day 7	Day 11	Day 15	TOTAL	% of Correct Classification
Day 0	14	0	0	0	0	14	100%
Day 3	0	14	0	0	0	14	100%
Day 7	0	0	13	1	0	14	92.86%
Day 11	0	0	2	10	2	14	71.43%
Day 15	0	0	0	5	9	14	64.29%
TOTAL	14	14	15	16	11	70	85.71%

**Table 4:** Classification table of factorial discriminant analysis with Leave-One-Out Cross Validation of linseed oil during ageing obtained after excitation set at 270 nm.

**Observed/** **Predicted**	**Day 0**	**Day 3**	**Day 7**	**Day 11**	**Day 15**	**TOTAL**	**% of Correct Classification**
Day 0	14	0	0	0	0	14	100%
Day 3	0	14	0	0	0	14	100%
Day 7	0	0	12	0	2	14	85.71%
Day 11	0	0	1	7	6	14	50%
Day 15	0	0	3	4	7	14	50%
TOTAL	14	14	16	11	15	70	77.14%

**Table 5:** Classification table of factorial discriminant analysis applied to the concatenated first PCS of the PCA applied to the emission and excitation spectra set at 270 and 450 nm, respectively.

Observed/ Predicted	Day 0	Day 3	Day 7	Day 11	Day 15	TOTAL	% of Correct Classification
Day 0	14	0	0	0	0	14	100%
Day 3	0	14	0	0	0	14	100%
Day 7	0	0	14	0	0	14	100%
Day 11	0	0	0	12	2	14	85.71%
Day 15	0	0	0	4	10	14	71.43%
TOTAL	14	14	14	16	12	70	91.43%

## Data Availability

Data is contained within the article.
